# Human Milk Sodium Monitoring Across the Continuum of Lactation: From Secretory Activation to Established Lactation

**DOI:** 10.3390/nu18162705

**Published:** 2026-08-19

**Authors:** Katsumi Mizuno, Tomoko Himi

**Affiliations:** 1 Department of Pediatrics, Showa Medical University, 1-5-8, Hatanodai, Shinagawa-ku, Tokyo 142-8666, Japan; 2 The Oketani Breastfeeding Management Research Institute, 20-8, Kikuicho, Shinjuku-ku, Tokyo 162-0044, Japan; himiko4716@gmail.com

**Keywords:** human milk, sodium, secretory activation, home monitoring, lactation support

## Abstract

**Background:** Human milk sodium (Na) concentration is a biomarker of lactation physiology and secretory activation, but conventional measurement methods are impractical for routine breastfeeding support. Portable Na analyzers may enable real-time home monitoring. **Objective:** The purpose of this study was to characterize longitudinal changes in human milk Na concentrations during the first 3 postpartum months and to evaluate the feasibility of longitudinal home Na monitoring throughout established lactation. **Methods:** Nine healthy lactating mothers intending to breastfeed were recruited at a breastfeeding management clinic in Tokyo, Japan, between August 2025 and December 2025. Participants performed home-based Na measurements from both breasts from birth through approximately 100 days postpartum, with the planned monitoring schedule defined as 90 postpartum days for adherence assessment. Each measurement required 2–3 drops of milk and was completed within 1 min. Longitudinal Na trajectories and Na elevation episodes were evaluated. **Results:** Na concentrations were highest immediately after birth and declined rapidly during the first postpartum week. Six of nine participants (66.7%) reached concentrations below 413.8 ppm within the first 7 postpartum days, and five of nine (55.6%) reached concentrations below 220 ppm by postpartum day 8. During established lactation, 62 breast-level Na elevation episodes were identified, with substantial interindividual variation in frequency and duration. Some episodes occurred without overt breast symptoms. Frequent home monitoring detected transient physiological fluctuations that would likely have been missed by conventional intermittent sampling. **Conclusions:** Longitudinal home milk Na monitoring was feasible in this small pilot cohort and enabled characterization of Na fluctuations from secretory activation through established lactation. These findings are preliminary and hypothesis-generating, and larger prospective studies are required to determine the clinical utility of longitudinal Na monitoring for breastfeeding assessment.

## 1. Introduction

Human milk provides optimal nutrition for newborn infants and contains bioactive components that support growth, immune development, and protection against infection [1]. Successful breastfeeding depends on timely secretory activation and the maintenance of adequate milk production throughout lactation. Secretory activation is the physiological transition from colostrum production to copious milk secretion. This process is accompanied by closure of mammary epithelial tight junctions, resulting in a rapid decline in human milk Na concentration. Accordingly, milk Na concentrations are relatively high in colostrum, decline rapidly during the transitional milk period as secretory activation progresses, and subsequently reach lower concentrations in mature milk [2,3,4,5]. Consequently, milk Na has long been recognized as an objective biomarker of lactation physiology and the establishment of breastfeeding [3,4,5,6,7,8].

Beyond the early postpartum period, elevated milk Na concentrations have also been associated with breast engorgement, milk stasis, mastitis, and maternal perceptions of insufficient milk supply [8,9,10,11]. Although these findings suggest that milk Na may reflect ongoing mammary gland function throughout lactation, its clinical application has remained limited because conventional measurement requires laboratory-based analysis.

Recent advances in portable Na analyzers have enabled rapid point-of-care and home-based assessment of milk Na concentrations [11,12,13,14]. Previous studies have demonstrated the feasibility of identifying secretory activation using handheld devices and have suggested that once-daily measurements adequately characterize early postpartum Na trajectories. However, these investigations primarily focused on early lactation, and the potential role of longitudinal Na monitoring during established lactation remains largely unexplored.

Emerging evidence also suggests that different Na thresholds may reflect distinct physiological stages of lactation. A threshold of 413.8 ppm has been associated with secretory activation [13], whereas our previous prospective study suggested that concentrations below 220 ppm may indicate transition to stable mature lactation [15]. Whether serial home monitoring of milk Na can provide clinically useful information beyond these physiological transitions has not been determined.

Therefore, the purpose of this study was to characterize longitudinal bilateral human milk Na concentrations from secretory activation through established lactation using serial home monitoring with a portable point-of-care analyzer. Unlike previous studies that focused primarily on secretory activation, the present study investigated whether continuous longitudinal home monitoring could identify transient Na fluctuations during established lactation, thereby providing new insight into the dynamic physiology of the lactating mammary gland.

## 2. Methods

### 2.1. Participants and Study Design

Nine healthy lactating mothers were enrolled in this prospective observational study. All nine mothers who were approached agreed to participate. Participants were recruited at the Oketani Breastfeeding Management Research Institute (Tomoko Himi), Tokyo, Japan, between 1 August 2025 and 25 December 2025. Eligible participants were healthy mothers who delivered a live infant, had no medical conditions known to interfere with lactation, and intended to breastfeed.

Participants collected milk samples from both breasts by manual expression before breastfeeding (foremilk) to standardize sampling, although previous work has reported no significant difference in Na concentration between foremilk and hindmilk [16]. Participants measured Na concentrations at home using a portable ion-selective electrode sodium analyzer (LAQUA Twin; HORIBA Ltd., Kyoto, Japan). To minimize within-day biological variability, participants were instructed to perform measurements between 11:00 AM and 1:00 PM whenever possible. Because only 2–3 drops of milk were required for each measurement, manual expression was sufficient for sample collection. Each measurement required 2–3 drops of milk and was completed within one minute. The device has previously been validated against laboratory-based inductively coupled plasma optical emission spectrometry for measurement of human milk Na concentrations [10,12].

Participants were asked to perform one home measurement session per day for 90 postpartum days, with Na concentrations measured separately in milk from both breasts. Measurements beyond postpartum day 90 were available for some participants but were not included when calculating adherence to the planned monitoring schedule. Participants who repeatedly discontinued measurements before the planned end of follow-up were considered to have withdrawn from the study. At each measurement, they recorded breastfeeding practices, milk expression, and breast-related symptoms. Na concentrations were recorded in parts per million (ppm), the native output of the analyzer (1 ppm ≈ 0.0435 mmol/L). Because the objective of this study was to evaluate the feasibility of home monitoring using a portable analyzer, Na concentrations are presented in ppm to reflect the values displayed by the device and to maintain consistency with our previous study using the same methodology [15]. Participants performed serial home measurements throughout the observation period, generally on a daily basis.

Milk oversupply tendency was classified retrospectively by the principal investigator (T.H.), a certified lactation consultant, based on maternal report of abundant milk production, frequent leakage between feedings, and infant satisfaction with unilateral feeds, recorded in the study diary at each home measurement session. Classification was performed prior to data analysis and was based on clinical records rather than quantitative measurement of 24-h milk production.

This prospective observational study was designed as a feasibility pilot study to evaluate the feasibility and practicality of longitudinal home Na monitoring and to generate hypotheses for future adequately powered prospective studies rather than to establish clinical effectiveness, consistent with the objectives of feasibility and pilot studies as described by Arain et al. [17].

### 2.2. Definitions and Outcomes

For the purposes of this study, full breastfeeding was defined as feeding with breast milk without infant formula after a period of mixed feeding. Exclusive breastfeeding from birth was distinguished from full breastfeeding established later during lactation. Based on Haramati et al. [13], Na concentrations < 413.8 ppm were considered indicative of secretory activation. Based on our previous longitudinal study, concentrations < 220 ppm were used as an exploratory marker of transition to stable mature lactation [15].

For each participant, established lactation was defined as beginning on the first postpartum day on which Na concentrations in both breasts were <220 ppm. Na elevation episodes were counted separately for each breast beginning after this participant-specific transition. An elevation episode was defined as one or more observed measurements > 220 ppm and was considered to end when a subsequent observed Na concentration was ≤220 ppm. Missing measurement days were treated as missing and did not, by themselves, terminate an ongoing episode or define a new episode.

The primary outcome was the longitudinal trajectory of milk Na concentrations during the observation period. Secondary outcomes included the timing of secretory activation, transition to mature lactation, and frequency of Na elevation episodes.

### 2.3. Descriptive and Exploratory Analysis

Early postpartum and established lactation periods were analyzed separately. Analyses of established lactation included measurements obtained from the participant-specific starting point defined above through postpartum day 80. Na concentrations were summarized using descriptive statistics, including the mean, standard deviation (SD), median, interquartile range (IQR), range, and coefficient of variation (CV).

Because this was a feasibility pilot study involving only nine participants, analyses were primarily descriptive and exploratory. Although repeated measurements were clustered within participants and breasts, inferential longitudinal analyses such as mixed-effects models or generalized estimating equations were not performed because of the limited number of independent participants. For exploratory participant-level analyses, mean Na concentrations during established lactation were compared between primiparous and multiparous mothers using the Mann–Whitney U test. Breast laterality was examined by comparing participant-level mean Na concentrations between the right and left breasts using the Wilcoxon signed-rank test. In addition, for each participant, the proportion of breast-level Na measurements exceeding 220 ppm during established lactation was calculated and compared between mothers with and without a tendency toward milk oversupply using the Mann–Whitney U test.

Data management, descriptive analyses, and exploratory nonparametric analyses were performed using SPSS version 25 (IBM Corp., Armonk, NY, USA).

### 2.4. Ethics Approval

The study was approved by the Ethics Committee for Human Subject Research of Showa Medical University (Approval No. 2023-217-A) on 12 January 2024. Written informed consent was obtained from all participants. The study was conducted in accordance with the Declaration of Helsinki.

## 3. Results

### 3.1. Baseline Maternal and Infant Characteristics (Table 1)

**Figure 1:**
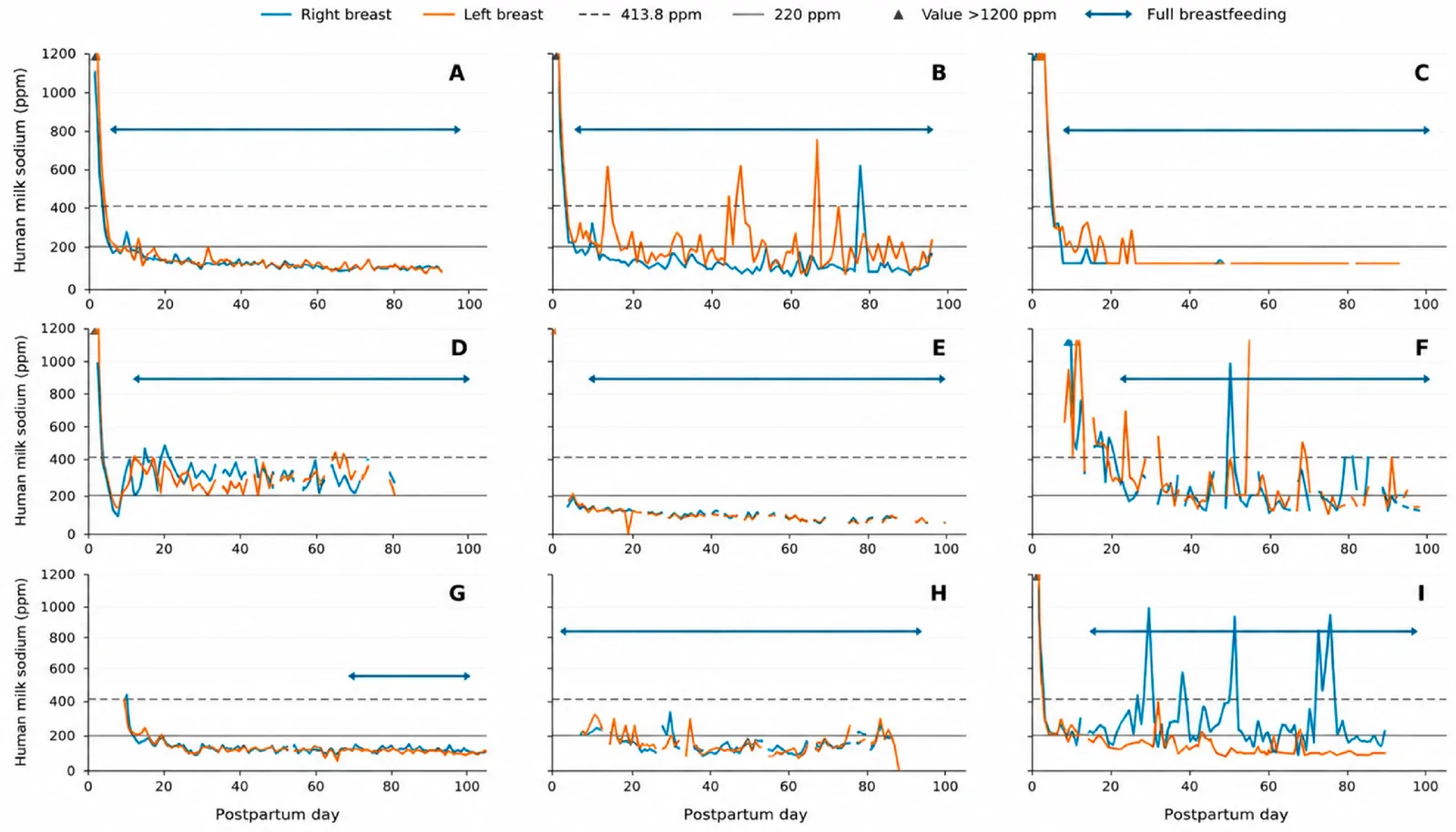
(**A**–**I**) individual longitudinal trajectories of breast-milk Na concentrations for Participants A–I, respectively. (**A**) Participant A; (**B**) Participant B; (**C**) Participant C; (**D**) Participant D; (**E**) Participant E; (**F**) Participant F; (**G**) Participant G; (**H**) Participant H; (**I**) Participant I. Individual longitudinal trajectories of bilateral human milk Na concentrations during the first 80–100 postpartum days. Na concentrations were measured separately in milk obtained from the right and left breasts. Horizontal reference lines indicate the proposed thresholds for secretory activation (413.8 ppm) and stable mature lactation (220 ppm). The horizontal bar labeled “Full breastfeeding” indicates the period during which the infant received breast milk without infant formula. Before the establishment of full breastfeeding, infants received mixed feeding consisting of breast milk and infant formula. Participants A and H were exclusively breastfed from birth.

Participants showed variation in breastfeeding patterns during the study period (Table 2 and Figure 1). Participants A and H were exclusively breastfed from birth, whereas the remaining participants initially received mixed feeding with breast milk and infant formula and subsequently transitioned to full breastfeeding. Full breastfeeding was established on postpartum days 4, 7, 15, 15, 21, 68, and 18 in Participants B, C, D, E, F, G, and I, respectively. Four participants (A, D, H, and I) showed a tendency toward milk oversupply. By the end of the study period, all nine participants had established full breastfeeding. Feasibility of home monitoring was assessed against the planned schedule of one measurement session per day for 90 postpartum days. Overall, 706 of 810 expected participant-days were completed (87.2%), corresponding to 1412 of 1620 expected breast-level measurements. Participant-level completion rates ranged from 73.3% to 100.0%. Two participants completed all 90 planned measurement days, while three discontinued monitoring before postpartum day 90.

**Table 1:** Baseline Characteristics of the Study Participants (n = 9).

Characteristic	Value
**Maternal characteristics**	
Maternal age, years, median (IQR)	32 (30–38)
Primiparous, n (%)	4 (44.4)
Multiparous, n (%)	5 (55.6)
Full breastfeeding at study completion, n (%)	9 (100)
Milk oversupply tendency, n (%)	4 (44.4)
**Infant characteristics**	
Gestational age at delivery, weeks, median (IQR)	39 (38–40)
Birth weight, g, median (IQR)	3022 (2830–3088)
Female infant, n (%)	5 (55.6)
Male infant, n (%)	4 (44.4)

Abbreviation: IQR, interquartile range. Full breastfeeding was defined as feeding with breast milk without infant formula. Participants A and H were exclusively breastfed from birth; the other participants established full breastfeeding after a period of mixed feeding.

**Table 2:** Maternal and Infant Characteristics of Individual Study Participants.

Participant	Age (ys)	Parity	GA (wk + d)	BW (g)	Sex	FB Day	Clinical Characteristics
A	32	Primi	38 + 3	3040	F	0	Oversupply tendency
B	40	Multi	40 + 2	3088	F	4	—
C	38	Primi	38 + 6	3022	M	7	—
D	28	Primi	39 + 2	2790	F	15	Oversupply tendency
E	30	Multi	39 + 1	2830	F	15	—
F	30	Primi	38 + 4	3076	F	21	—
G	40	Multi	35 + 0	2679	M	68	Emergency cesarean delivery for placenta previa; infant admitted to the NICU for 10 days
H	36	Multi	41 + 0	3000	M	0	Oversupply tendency
I	36	Multi	40 + 2	3414	M	18	Oversupply tendency

**Abbreviations:** Primi, primiparous; Multi, multiparous; GA, gestational age; BW, birth weight; F, female; M, male; FB, full breastfeeding; NICU, neonatal intensive care unit. FB day indicates the postpartum day on which full breastfeeding was established. Participants A and H were exclusively breastfed from birth; the remaining participants received mixed feeding before establishing full breastfeeding. Oversupply indicates a tendency toward milk oversupply.

### 3.2. Secretory Activation and Transition to Stable Mature Lactation

Na concentrations were highest immediately after birth and generally declined during the early postpartum period (Figure 1). Using the threshold proposed by Haramati et al. [13], six of nine participants (66.7%) reached Na concentrations below 413.8 ppm within the first 7 postpartum days. Na concentrations continued to decline thereafter, and five of nine participants (55.6%) reached concentrations below the exploratory threshold of 220 ppm by postpartum day 8. All nine participants eventually reached concentrations below 413.8 ppm and subsequently below 220 ppm, although the timing varied among participants. The first occurrence of Na concentrations below 413.8 ppm ranged from postpartum days 3 to 13, whereas the first occurrence below 220 ppm ranged from postpartum days 5 to 24. The longitudinal trajectories demonstrated that decreases in milk Na concentration occurred as a continuous process rather than a single physiological event. Following attainment of concentrations below 413.8 ppm, further reductions toward 220 ppm were observed before concentrations stabilized during established lactation.

### 3.3. Na Dynamics During Established Lactation (Figure 2)

To account for interindividual variation in the timing of transition to mature lactation, established-lactation analyses began on the first postpartum day on which Na concentrations in both breasts were <220 ppm for each participant and continued through postpartum day 80 (Figure 1). Participant-specific starting points ranged from postpartum day 4 to day 35. A total of 1110 breast-level measurements were included in these analyses. The participant-level mean Na concentration was 187.14 ± 66.33 ppm, with a median (IQR) of 159.86 (138.15–232.14) ppm. The participant-level mean coefficient of variation was 33.90 ± 17.27% (range, 15.37–64.36%), indicating substantial variability despite transition to established lactation.

Three exploratory subgroup comparisons were performed at the participant level. Participant-level mean Na concentrations did not differ significantly between primiparous (n = 4) and multiparous (n = 5) mothers (Mann–Whitney U = 15.0, exact *p* = 0.286). The participant-level proportion of Na measurements exceeding 220 ppm did not differ significantly between mothers with and without a tendency toward milk oversupply (median, 22.5% vs. 5.8%; Mann–Whitney U = 15.0, exact *p* = 0.286; Figure 3). No significant difference in participant-level mean Na concentrations was detected between right and left breasts (median, 152.9 vs. 155.9 ppm; Wilcoxon signed-rank test, exact *p* = 1.000). All three comparisons were underpowered given the small sample size and should therefore be interpreted as hypothesis-generating rather than as evidence for the absence of associations.

**Figure 2:**
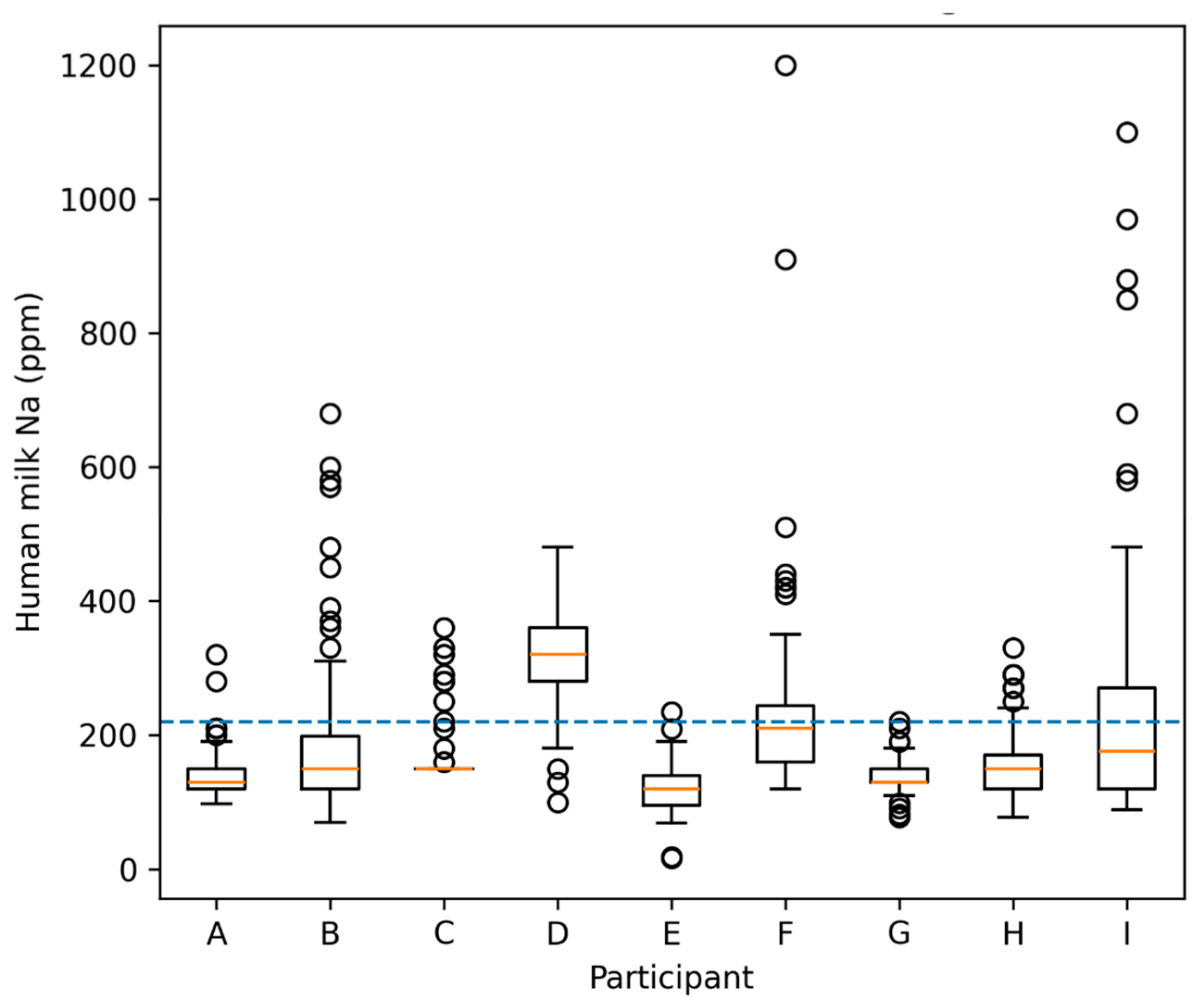
Distribution of human milk Na concentrations during established lactation. For each participant, the analysis period began on the first postpartum day on which Na concentrations in both breasts were <220 ppm and continued through postpartum day 80. Boxplots represent the distribution of breast-level Na measurements for each participant. The central line indicates the median, boxes represent the interquartile range (IQR), whiskers indicate values within 1.5 × IQR, and individual outliers are shown as points. The dashed horizontal line indicates the exploratory threshold of 220 ppm.

**Figure 3:**
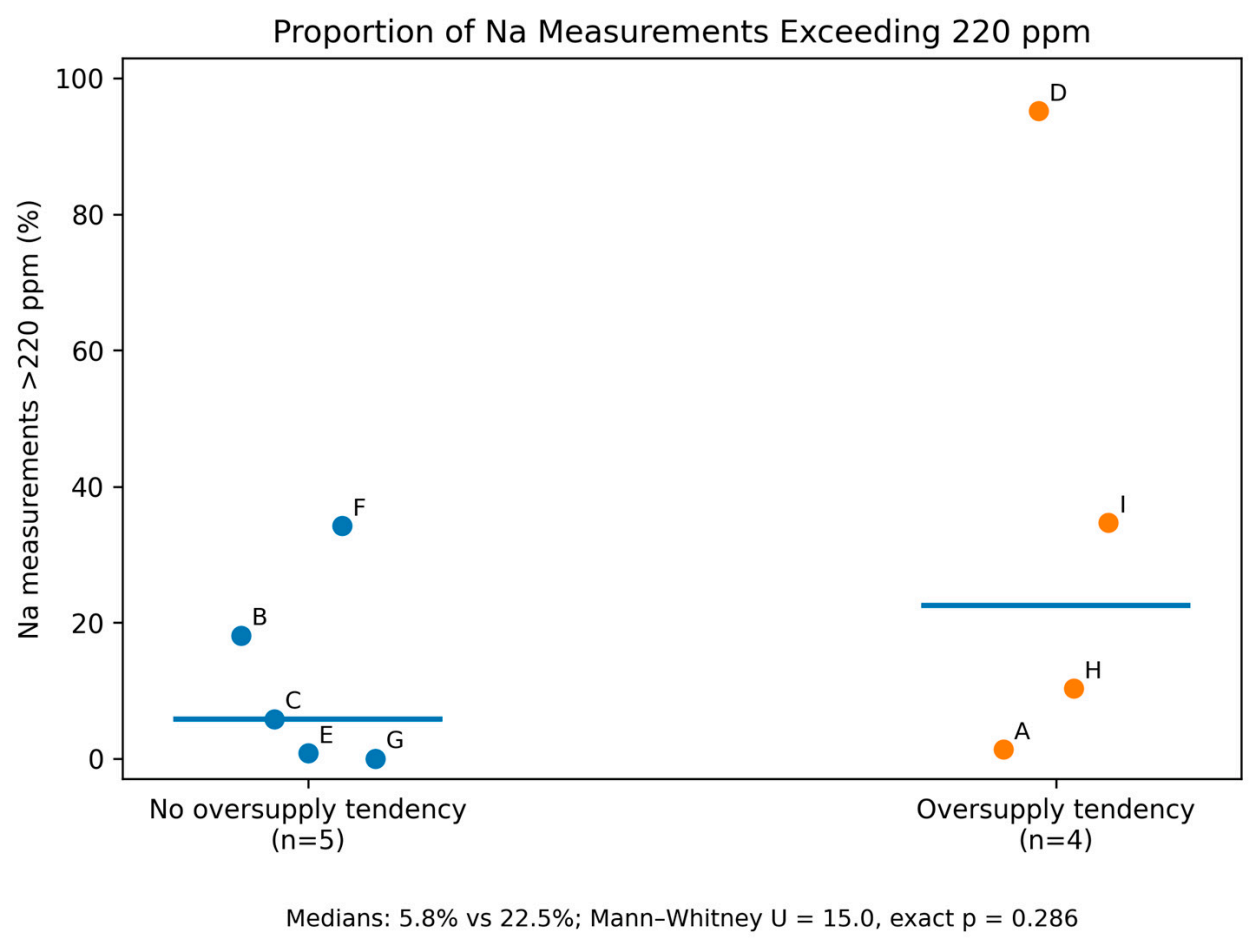
Proportion of Na measurements exceeding 220 ppm during established lactation according to maternal milk oversupply tendency. For each participant, established lactation began on the first postpartum day on which Na concentrations in both breasts were <220 ppm. Each point represents one participant. Horizontal lines indicate group medians. The median proportion was 22.5% in mothers with an oversupply tendency and 5.8% in those without an oversupply tendency (Mann–Whitney U = 15.0, exact *p* = 0.286).

### 3.4. Na Elevation Episodes and Subsequent Breastfeeding-Related Responses

Following the participant-specific transition to established lactation, a total of 62 breast-level Na elevation episodes were identified across the study cohort. Episode frequency varied markedly among participants, ranging from no episodes in Participant G to 14 episodes in Participants F and I. The pattern of Na elevation also differed substantially among participants; some experienced brief recurrent elevations, whereas others, particularly Participant D, showed prolonged periods of Na concentrations above 220 ppm.

Some Na elevation episodes were brief and isolated, whereas others persisted for several days or recurred during the observation period. Some episodes were accompanied by breast discomfort or a sensation of milk stasis, whereas others occurred in the absence of subjective symptoms. No participant developed clinical mastitis during the study period.

In several instances, participants reported increasing breastfeeding frequency, performing additional milk expression, or modifying breastfeeding practices after an increase in milk Na concentration was identified, and subsequent declines in Na concentration were observed in some of these instances. However, breastfeeding-related responses were not systematically recorded for every Na elevation episode; therefore, these temporal associations could not be reliably quantified and are presented only as anecdotal, hypothesis-generating observations.

## 4. Discussion

The present study demonstrates that longitudinal home monitoring of human milk Na concentration is feasible and provides longitudinal physiological information throughout the continuum of lactation. Beyond its traditional role as a biomarker of secretory activation, serial Na monitoring may provide additional information regarding ongoing lactation physiology.

Consistent with previous reports [2,5], milk Na concentrations generally declined during the early postpartum period. Six of nine participants (66.7%) reached Na concentrations below 413.8 ppm within the first 7 postpartum days, consistent with secretory activation as described by Haramati et al. [13]. Five of nine participants (55.6%) subsequently reached concentrations below the exploratory threshold of 220 ppm by postpartum day 8. However, the timing of these transitions varied considerably among participants: the first occurrence of Na concentrations below 413.8 ppm ranged from postpartum days 3 to 13, whereas the first occurrence below 220 ppm ranged from postpartum days 5 to 24. These findings suggest that the transition from secretory activation to established lactation is a gradual process with substantial interindividual variation rather than a single uniform physiological event. Whereas a threshold of approximately 413.8 ppm has been associated with secretory activation, further decline toward the exploratory threshold of 220 ppm may reflect progression toward a stable mature lactation state.

The most novel finding of this study was the identification of 62 transient Na elevation episodes during established lactation. These episodes frequently occurred in the absence of overt breast symptoms and would likely have been missed using conventional intermittent sampling. The dense longitudinal dataset generated by near-daily home monitoring demonstrated that milk Na fluctuations remained common even after mature lactation was established. Exploratory analyses did not identify significant associations with parity or milk oversupply tendency, although the small sample size limits interpretation. These findings suggest that milk Na may reflect dynamic changes in mammary gland physiology.

In several instances, declines in Na concentration were observed after participants reported increasing breastfeeding frequency or milk expression. However, breastfeeding-related responses were not systematically recorded for all Na elevation episodes, and these observations were therefore not quantitatively analyzed. Because this was an observational pilot study without a controlled intervention, these temporal observations should be considered anecdotal and hypothesis-generating and should not be interpreted as evidence of therapeutic effectiveness. Spontaneous physiological recovery or regression toward baseline cannot be excluded. Nevertheless, these observations suggest that serial Na monitoring may provide objective physiological information to complement maternal symptoms and clinical assessment. When a marked increase in milk Na concentration was detected, participants were informed that the finding might reflect transient milk accumulation within the mammary gland and were encouraged to review breastfeeding practices, including increasing breastfeeding frequency or expressing milk when appropriate.

Although all participants were highly motivated to breastfeed, the time required to establish full breastfeeding varied considerably among participants. Breastfeeding-related breast and nipple problems are particularly common during the early postpartum period and may persist for several weeks after birth [18,19]. Therefore, longitudinal monitoring throughout the first three postpartum months provided an opportunity to characterize dynamic changes in mammary gland physiology during a period when breastfeeding difficulties commonly occur. Notably, all nine participants had established full breastfeeding by the end of the observation period, and none developed clinical mastitis. For context, the 2015 National Infant and Young Child Nutrition Survey in Japan reported that 54.7% of infants received breast milk without infant formula at 3 months of age [20]. Although these observations raise the possibility that repeated physiological feedback from home Na monitoring, together with individualized breastfeeding support, may have helped participants recognize and respond to changes in milk removal, the present uncontrolled pilot study cannot determine whether Na monitoring contributed to the establishment or maintenance of full breastfeeding or to the absence of mastitis. These observations should therefore be considered hypothesis-generating and warrant evaluation in larger controlled prospective studies.

Previous studies have associated elevated milk Na concentrations with delayed secretory activation, mastitis, and maternal perceptions of insufficient milk supply [5,6,7,8,9,10,11]. Our findings extend these observations by suggesting that home Na monitoring may also facilitate earlier recognition of transient disturbances in milk removal before clinically significant breastfeeding problems become apparent [21,22].

One participant with apparent milk oversupply (Participant D) demonstrated prolonged Na elevation rather than frequent discrete elevation episodes, with Na concentrations remaining above 220 ppm for extended periods in both breasts. Importantly, despite these sustained Na elevations, this participant successfully established full breastfeeding. This observation highlights that milk Na concentration should not be interpreted in isolation as an indicator of breastfeeding adequacy or used as a stand-alone measure to guide breastfeeding support. Rather, Na monitoring, if clinically validated, may be most appropriately considered as complementary physiological information alongside breastfeeding patterns, maternal symptoms, milk production and removal, and clinical assessment.

Our previous study demonstrated that elevated milk Na concentrations are associated with impaired mammary gland integrity in mothers with ductal obstruction, supporting the hypothesis that sustained or transient Na elevations may reflect disruption of epithelial barrier function rather than insufficient milk production alone [15]. Not all mothers with milk oversupply demonstrated prolonged Na elevation, suggesting that oversupply itself is unlikely to be the direct cause. Rather, a transient imbalance between milk production and milk removal may contribute to these Na elevations. This hypothesis requires confirmation in larger prospective studies. A major strength of this study is the exceptionally dense longitudinal dataset comprising more than 1100 repeated measurements collected over approximately three months. Despite occasional missed measurements and incomplete follow-up in some participants, all nine participants were able to perform serial home Na measurements over an extended postpartum period. This experience supports the feasibility of longitudinal home Na monitoring in this selected cohort. The high temporal resolution enabled characterization of short-term physiological fluctuations that would likely have been missed by conventional intermittent sampling.

This study has several limitations that should be considered when interpreting the findings. First, this was a single-center pilot study with a small sample size (n = 9). All analyses, including those of Na dynamics during established lactation, the timing of transition to stable mature lactation, and the exploratory subgroup comparisons, are therefore preliminary and should be replicated in larger prospective cohorts before any clinical conclusions are drawn. Second, participants were recruited from a single specialized breastfeeding management clinic and were highly motivated to breastfeed. This selection may limit the generalizability of the observed breastfeeding outcomes and adherence rates (87.2% completion of expected measurement days; three of nine participants discontinued before postpartum day 90) to the general postpartum population. Third, breastfeeding frequency, milk volume, infant milk intake, and maternal perceptions of milk supply were not systematically quantified, limiting interpretation of the physiological mechanisms underlying individual Na elevation episodes. Fourth, the exploratory 220 ppm threshold was derived from our previous longitudinal study and requires external validation in larger independent cohorts before it can be considered a clinically meaningful cutoff. Fifth, attrition bias cannot be excluded. Not all participants completed the planned 90-day follow-up because of repeated missed measurements, and missing measurement days were treated as missing in the analyses. The impact of non-random dropout on the observed Na trajectories is unknown. Sixth, the exploratory subgroup comparisons reported in Section 3.3 (parity, milk oversupply tendency, and between-breast asymmetry) were each based on a sample size of n = 4 vs. n = 5 and were therefore underpowered. Non-significant results should not be interpreted as evidence for the absence of associations, but rather as hypothesis-generating findings requiring validation in larger cohorts. Seventh, the temporal association between Na elevation episodes and changes in breastfeeding frequency or milk expression was not systematically recorded for every episode. Therefore, the impression that “many” episodes resolved after increased breastfeeding or expression could not be reliably quantified and is presented as anecdotal and hypothesis-generating only. This observation should not be interpreted as evidence of a treatment-responsive pattern in this pilot study.

## 5. Conclusions

In conclusion, serial human milk Na monitoring is a feasible approach for longitudinal assessment of lactation physiology and may provide an objective method for monitoring changes throughout the continuum of lactation. However, this exploratory pilot study does not establish clinical effectiveness, predictive value, or improvement in breastfeeding outcomes. Larger prospective studies are required to validate the proposed Na thresholds and determine the clinical utility of longitudinal Na monitoring for breastfeeding assessment.

## Data Availability

The original contributions presented in this study are included in the article. Further inquiries can be directed to the corresponding author.
